# DNN-assisted waveguide width extraction via optical measurement of a single low-order Mach-Zehnder interferometer

**DOI:** 10.1038/s41598-026-41085-2

**Published:** 2026-03-05

**Authors:** Fuling Wang, Hao You, Xiao Xu, Liuge Du, Chonglei Sun, Jia Zhao

**Affiliations:** School of Information Science and Engineering, Shandong University, Qingdao, 266237 China

**Keywords:** Waveguide width extraction, Mach-Zehnder interferometers, Deep neural networks, Silicon photonics, Engineering, Optics and photonics, Physics

## Abstract

An accurate waveguide width extraction methodology is demonstrated by developing a deep-learning neural network (DNN) framework synergized with a single low-order Mach-Zehnder interferometer (MZI). Utilizing its intrinsic capacity for complex nonlinear capture, the proposed DNN achieves a simulation-to-prediction accuracy of 0.15 nm on a test dataset, establishing a robust geometry mapping. By leveraging fabrication tolerance analysis and group-index estimation from C-band MZI interference spectra, our method directly extracts the as-fabricated waveguide width via a pre-trained DNN. Experimental validation on 30 devices demonstrates an experimental estimation accuracy of 3.28 nm when compared to SEM measurements. Additionally, the system operates with a single low-order MZI featuring a reduced arm-length difference of 26.672 μm, while the conventional ones exceeding 100 μm. The superior precision and miniaturization capability make our approach an effective strategy for fabrication monitoring and high-density photonic circuit characterization.

## Introduction

The rapid expansion of silicon photonics has emerged to satisfy the insatiable demand for data storage and communication in data centers, high performance computing and quantum computing systems^1–5^. Owing to high index contrast and high mode confinement, silicon-on-insulator (SOI) waveguides enable compact integration of photonic devices. However, the optical performance of SOI waveguides is highly sensitive to geometric parameters, which often deviate from design values due to fabrication imperfections (e.g., lithography variations and etching nonuniformity). Due to synergistic interaction of high optical sensitivity and a greater absolute variation, waveguide width has a more significant impact on optical mode confinement, propagation loss, coupling efficiency, and dispersion engineering^6^. Efficient extraction of this parameter enables wafer-level statistical process control, facilitates performance prediction of photonic integrated circuits, and provides essential feedback for fabrication optimization.

Conventional methods for waveguide linewidth characterization, such as scanning electron microscopy (SEM) and atomic force microscopy (AFM), can provide direct and high-resolution measurements^7–9^. But these techniques require lengthy operation time and costly equipment. Once the optical device is fabricated and delivered to the customer, AFM/SEM testing cannot be performed without destroying the cladding layer on the chip. In contrast, optical measurement techniques extract the geometric parameters by directly probing the optical behavior of the fabricated structures. A promising strategy involves placing reference photonic elements—such as Mach-Zehnder interferometers (MZIs) or microring resonators^10–12^—in close proximity to the target devices. Under the assumption that nearby devices share identical fabrication conditions and hence similar waveguide dimensions, the optical responses of these reference structures (e.g., resonance wavelength shifts or interference patterns) can be measured and correlated to geometric variations via induced effective index changes. By analyzing these spectral features, the waveguide linewidth can be accurately inferred, thereby enabling non-contact and in-situ characterization of photonic components without compromising device integrity.

Recent studies have demonstrated the possibility to extract effective index *n*_*e*_ by conducting the combined analysis to the wavelength spectra of low-order and high-order MZIs^11,13^. The low-order MZIs are used to isolate phase-derived *n*_*e*_ values, while high-order MZIs enhance sensitivity to dispersion-induced spectral shifts. By applying a polynomial model to correlate *n*_*e*_ with waveguide dimensions, this approach enables accurate determination of waveguide geometry^6^. However, the methods described above face two key challenges. On the one hand, employing multiple devices increases the footprint and multiple measurements, which in turn introduce variability and uncertainty. On the other hand, the geometry model based on finite-order mathematical fitting presents both simplicity and interpretability. Nevertheless, it faces inherent limitations in nonlinear adaptability, particularly near critical points like cutoff widths or under strong dispersion, where complex, high-order fits are required, increasing the risk of overfitting and computational burden. Furthermore, effectively modeling systems with multiple parameters amplifies these challenges, as the complexity of polynomial representations grows prohibitively. Recently, the development of DNN methods in nanophotonic and optical communication networks^14–16^ has leveraged their intrinsic capacity to capture complex nonlinear relationships through layered transformations. This establishes robust geometry models that mitigate overfitting and maintain reliability in out-of-distribution regimes. Critically, DNNs efficiently scale to multiple inputs—especially in multi-parameter scenarios, where flexible input layer expansion enables adaptive processing. The trained neural network thus functions as a “black box,” translating optical measurements into geometric parameters with high fidelity.

Concentrating on the challenges of multiple devices and geometry model in geometric parameter extraction, we utilizing a DNN algorithm, accompanied by a single low-order MZI to accurately extract the waveguide width of a SOI waveguide. Leveraging its intrinsic capacity for complex nonlinear capture, the DNN achieves a model mapping accuracy of 0.15 nm and establishes a robust geometry model crucial for the efficient design of modern photonic devices. By considering fabrication tolerance and applying a rough evaluation of the group index, we can accurately determine the effective index using the C-band interference troughs positions from a single low-order MZI. By mapping the effective index values into our pre-trained DNN, the waveguide widths of 30 fabricated devices can be accurately extracted. The experimental estimation accuracy when compared to SEM measurements, is as high as 3.28 nm. Moreover, our system employs a single low-order MZI with a significantly reduced arm-length difference of 26.672 μm, which is conducive to achieving high integration of silicon photonic devices. The proposed strategy not only complements traditional metrology tools but also paves the way for fabrication monitoring, particularly in process development and stabilization. By delivering accurate waveguide geometry feedback within minutes of wafer unloading, our method enables rapid adjustment of lithography or etching parameters, significantly accelerating the process optimization cycle compared to offline SEM/AFM techniques. While full integration into a production line requires further engineering, the speed, accuracy, and minimal footprint of our approach provide a crucial technological foundation for implementing real-time, data-driven process control in next-generation photonic integrated circuits.

## Methods

MZI exploits the interference between split and recombined light beams, are particularly sensitive to the geometric properties of the waveguides that form the interferometer arms^17–19^. Even small width variations can easily cause significant performance degradation, including shifts in the interference pattern, signal loss, and reduced device efficiency.

**Fig. 1:**
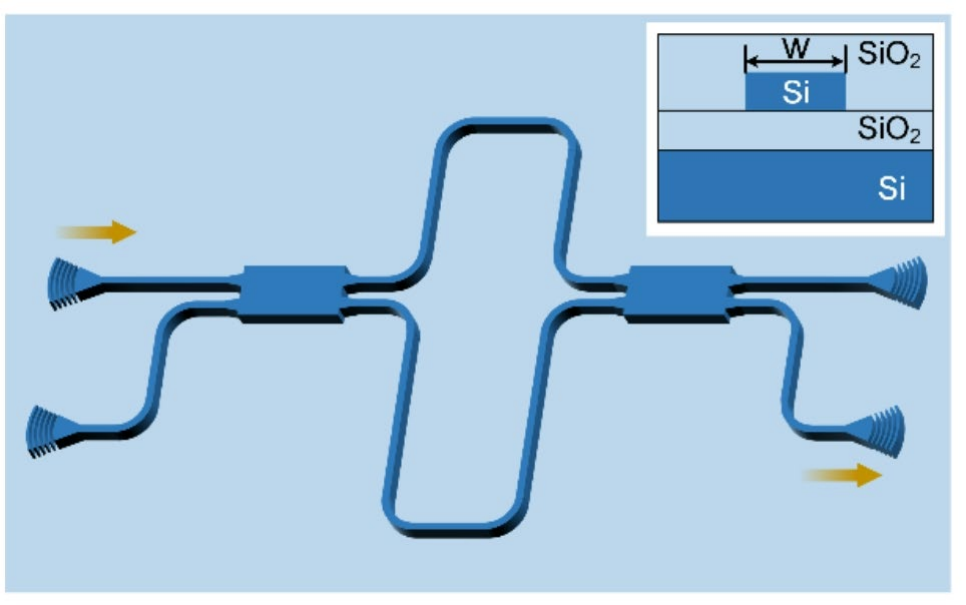
The schematic of MZI structure used for extracting waveguide width. Insert section: The cross-section of the oxide-clad Si waveguide.

The effective index of a SOI waveguide at a given wavelength is governed by material dispersion and geometric parameters, notably the waveguide width (*w*). To enable precise *w* extraction, an asymmetric MZI architecture was engineered as shown in Fig. 1. The cross-section of the oxide-clad Si waveguide is illustrated in the insert. To maintain single-mode condition, the fully etched silicon strip waveguide designed with a width *w* of 430 nm fabricated on a 220-nm-thick silicon device layer. The two interferometric arms of the MZI exhibit structure uniformity, with the sole distinction being the length of the straight waveguides at vertical part. Optical splitting/combining is achieved via symmetric 2 × 2 multimode interference (MMI) couplers. MMIs are relatively tolerant to fabrication variations in the multimode waveguide width and their symmetric design aims to minimize relative phase imbalance between the arms.

This design minimizes parasitic phase contributions from geometric or modal mismatches. Consequently, the MZI spectral response is principally governed by the intentionally introduced arm-length difference (*ΔL*) and the wavelength-dependent effective refractive index dispersion (*n*_*e*_*(λ)*), as expressed in the interference condition:

1 $$\Delta \varphi (\lambda )=\frac{{2\pi }}{\lambda } \cdot \Delta L \cdot {n_e}(\lambda ),$$.

where *Δφ* denotes the cumulative phase shift difference between the interferometric arms. When the phase difference is an odd multiple of π, destructive interference occurs at the cross port, corresponding to complete power annihilation in ideal interference conditions:

2 $$(2m\,+\,1) \lambda_{\mathrm{des}}\,=\,2{n_e}(\lambda_{\mathrm{des}})\Delta L,({\mathrm{m}}\,=\,0,{\mathrm{1}},{\mathrm{2}} \ldots )$$

where *λ*_*des*_ is destructive interference wavelength, *m* is the interference order, *n*_*e*_*(λ*_*des*_*)* is effective index of the waveguide at that wavelength. The periodic response of the MZI arises from the integer selection of its interference order. The free spectral range (FSR) of the MZI is defined as the difference between two destructive peaks:

3 $$FSR=\frac{{\lambda _{{{\mathrm{des}}}}^{2}}}{{{n_g}\Delta L}},$$

where *n*_*g*_ is the group index of the waveguide.

The change of effective index will lead to the drift of wavelength of the interference peak. If we know *λ*_*des*_ and *m*, we can calculate *n*_*e*_*(λ*_*des*_*)*. Leveraging the relationship between waveguide geometry and its effective index enables indirect but reliable waveguide width estimation.

### Effective index extraction

**Fig. 2:**
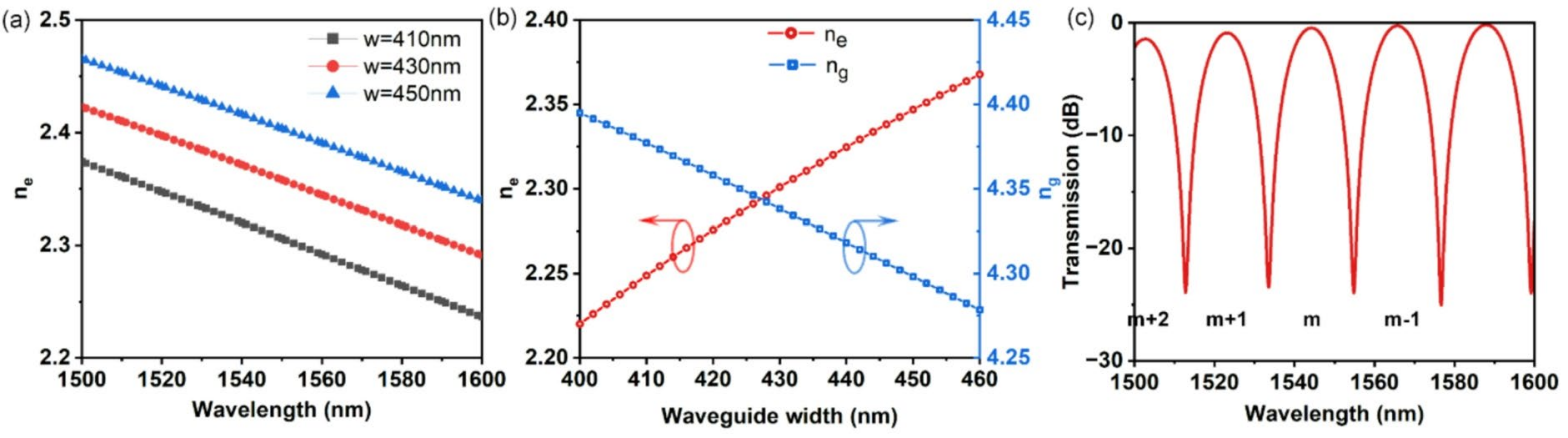
(**a**) The simulated effective index of different wavelength with several waveguide width. (**b**) The effective index and group index as functions of waveguide width. (c) Simulated transmission spectrum of MZI cross port.

In the transmission spectrum of the MZI, the positions of the peaks and trough give information about the effective index *n*_*e*_. According to Eqs. (2) and (3), low-order MZI generates interference fringes with wider wavelength spacing owing to their reduced *ΔL*. This expanded FSR enhances spectral resolution by minimizing fringe overlap and measurement uncertainty. However, a smaller *ΔL* also corresponds to a lower interference order *m*, which reduces the sensitivity of the interference wavelength to changes in the effective index, as seen from the derivative d*λ*_*des*_/d*n*_*e*_ ∝*ΔL*^20,21^. Therefore, selecting *ΔL* involves a trade-off between achieving a wide FSR and maintaining adequate sensitivity to fabrication-induced index variations. To operate within the C-band (around 1550 nm) and balance these factors, an interference order of *m* = 39 was chosen. This corresponds to a *ΔL* of 26.672 μm, calculated from Eq. (2) using the designed waveguide width (430 nm) and its simulated effective index. This design provides an FSR of approximately 21.6 nm, which is sufficiently wide to keep interference troughs well-separated across the C-band. Crucially, this wide separation is key to robustly resolving the interference-order ambiguity when the spectrum shifts due to fabrication variations, as explained in the subsequent paragraph. Additionally, the smaller *ΔL* naturally leads to a more compact device footprint.

Considering the ± 20 nm fabrication tolerance for 430-nm-wide waveguides in silicon photonics mass production, we conducted 2D mode analysis to simulate *n*_*e*_ of different wavelengths with the waveguide widths of 410 nm, 430 nm and 450 nm. The results are plotted in Fig. 2(a). According to Eq. (2), the 20 nm fabricated variation of *w* will present 14.90 nm wavelength shift. The simulated transmission spectrum of the designed MZI is shown in Fig. 2(c). The interference order *m* + 2, *m* + 1, … for MZI are marked. The 39th -order interference trough of the designed MZI occurs in the C-band (1535–1560 nm) at a wavelength of 1554.81 nm, with a FSR of 21.56 nm. Knowing the position of this interference trough in the C-band, it is theoretically possible to infer the effective index with the decided interference order. The simulated *n*_*e*_ and *n*_*g*_ with varying waveguide width at a wavelength of *λ* = 1554.81 nm are shown in Fig. 2(b). The sensitivity of the effective index of the designed waveguide (*w* = 430 nm) to its width is calculated as:

4 $$\frac{{\partial {{\mathrm{n}}_e}}}{{\partial w}}=0.002458\;n{m^{ - 1}}.$$

The fabricated variation of 430 nm waveguide is ± 20 nm, utilizing the *n*_*e*_ sensitivity of *w*, we can calculate the variation of *n*_*e*_:

5 $$\Delta {n_e}=\frac{{\partial {n_e}}}{{\partial w}} \cdot \Delta w=0.098328.$$

The designed path-length difference (*ΔL* = 26.672 μm) exceeds the critical value *λ/Δn*_*e*_ (15.81 μm). Here, the critical value *λ*/*Δn*_*e*_ represents the maximum allowable arm-length difference to ensure a unique interference order within one Free Spectral Range. *λ* is the operating wavelength (1554.81 nm), and *Δn*_*e*_ is the maximum effective index shift (0.098328) induced by the ± 20 nm width tolerance. When the actual *ΔL* exceeds this value, the fabrication-induced wavelength shift can span more than one FSR. Consequently, a single observed interference trough may correspond to two possible orders (e.g., a blueshifted order *m* or a redshifted order *m* + 1), creating ambiguity resolved by subsequent group-index analysis. The difficulty of analyzing a measured spectra lies in accurately guessing what particular interference trough position corresponds to a particular interference order. From Fig. 2(b), we can observe that when the fabricated waveguide width exceeds the design value (*Δw* > 0), the effective refractive index increases, leading to a redshift of the MZI spectrum. Conversely, when the waveguide width is smaller than designed (*Δw* < 0), *n*_*e*_ decreases, resulting in a blueshift of the MZI spectrum. If we detect an interference trough with a wavelength shorter than 1554.81 nm (*w* = 430 nm) in the C-band, it could ambiguously originate from either a redshifted (*m + 1*)-th order interference, or a blueshifted *m*-th order interference. To resolve this interference-order ambiguity, the group refractive index must be considered. While low-order MZI-based *n*_*g*_ extraction lacks high precision, it enables a qualitative judgment of width deviations:

1. If n^extracted^_g_ > n^design^_g_: Indicates Δ*w* < 0 (waveguide narrower than design), implying the observed trough corresponds to a blueshifted *m*-th order trough.
2. If n^extracted^_g_  < n^design^_g_: Suggests Δ*w* > 0 (waveguide wider than design), meaning the trough arises from a redshifted (*m + 1*)-th order trough.

By leveraging the *n*_*g*_-dependent correlation between fabrication deviations and spectral shifts, the interference order *m* can be unambiguously determined, yielding a unique solution for *n*_*e*_.

### Accurate *n*_*e*_*(λ*_*des*_*)*-to-*w* mapping

Because silicon waveguides are very dispersive, *n*_*e*_ varies with both wavelength (*λ*) and waveguide width (*w*). To establish the *n*_*e*_*(λ*_*des*_*)* -to-*w* mapping, a DNN is trained to predict the waveguide width *w* from the effective index at a certain wavelength. Training data is generated through finite-element method (FEM) simulations with the waveguide width and wavelength ranges set as *w* = 400 ~ 500 nm and *λ* = 1500 ~ 1600 nm, with 101 and 61 sampling points for each parameter, i.e., totally 6161 samples in the whole database. The dataset was partitioned with 80% (4928 samples) for training and 20% (1233 samples) for testing. The FEM-simulated dataset used to train and test the DNN in this study is available from the corresponding author upon reasonable request.

**Fig. 3:**
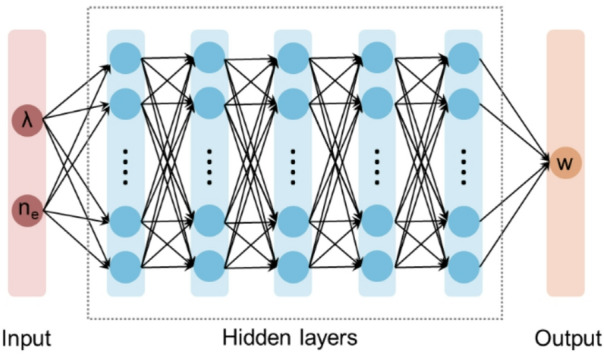
Architecture of the DNN.

We implement a fully connected network of seven layers, as shown in Fig. 3. Its architecture is denoted as 2-10-50-100-50-10-1, with the numerical values indicating the number of units in each layer. The ReLU activation function is uniformly applied for each DNN layer, for its superior performance in accelerating the network convergence. A learning rate of 10^− 3^ was selected to guarantee the best training speed and accuracy. The network underwent 1000 training epochs to minimize the mean absolute error (MAE) loss function:

6 $${\mathrm{los}}{{\mathrm{s}}_T}=\frac{1}{n}\sum\limits_{{i=1}}^{n} {(w_{{simu}}^{{(i)}} - w_{{pred}}^{{(i)}}} ),$$

**Fig. 4:**
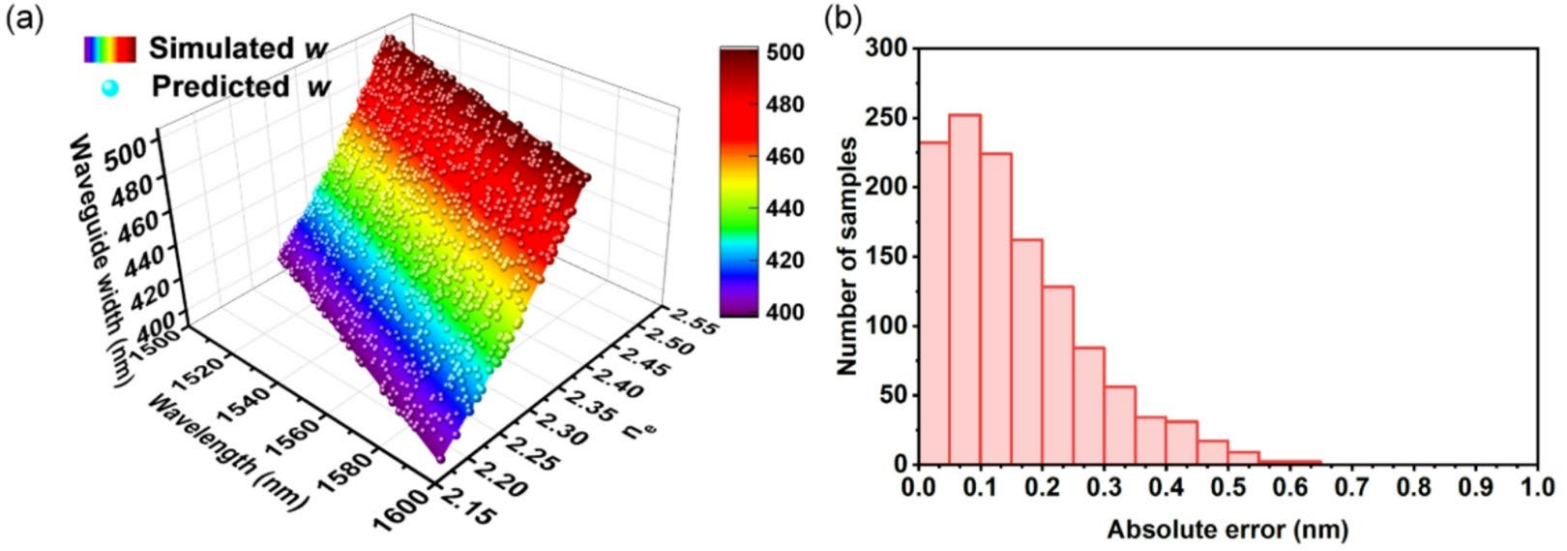
(**a**) The mapping performance of the DNN. (**b**) The distribution for MAE of the DNN predictions.

where *w*_*simu*_ and *w*_*pred*_ correspond to the simulated and predicted waveguide widths, respectively. Figure 4(a) plots the mapping performance, overlaying the DNN-predicted values (color-mapped spheres) on to the FEM-simulated *n*_*e*_*(λ*_*des*_*)*-*w* surface. We can see that the predicted points are in spatial alignment with the theoretical surface, indicating the capability of DNN to capture the *n*_*e*_*(λ*_*des*_*)*-to-*w* relationship. The statistical distribution of the absolute error in the DNN predictions is plotted in Fig. 4(b), where over 93% of samples achieve absolute errors < 0.35 nm, which confirms exceptional accuracy within the majority of the operational parameter space. The neural network, with an MAE of 0.15 nm across the test set, also demonstrates sub-nanometer precision in predicting waveguide widths. This result indicates a model prediction accuracy of 0.15 nm within the simulated parameter space, confirming the DNN’s precise learning of the underlying physical relationship.

For comparative analysis, we performed conventional polynomial fitting on the same simulation dataset. A second-order polynomial fit achieved a mean absolute error of 0.27 nm in width prediction, while a third-order polynomial reduced this error to 0.12 nm. Remarkably, DNNs autonomously learn dispersion relationships through nonlinear fitting yet achieve comparable accuracy to third-order polynomials—demonstrating computational intelligence surpassing traditional methods. This advantage is particularly pronounced in scenarios involving cut-off widths, strong dispersion regimes, and multi-parameter modeling—domains where polynomial approaches face fundamental limitations due to their inability to capture abrupt nonlinear transitions and complex variable interdependencies.

Functioning as a black box, the trained deep learning framework establishes a deterministic mapping between the wavelength-dependent effective index (*n*_*e*_*(λ*_*des*_*)*) and waveguide geometry (*w*), enabling accurate and reproducible waveguide width extraction from spectral data.

## Experimental results

In order to verify the effectiveness of this method, we fabricated the MZI devices and extracted the waveguide widths based on the test spectra. The devices were fabricated on a 220 nm-thick silicon device layer with SiO₂ cladding. Considering the fabricated waveguide width variation of ± 20 nm, we have no-pre-estimate of the nominal value of *w*, so that they can be any value under the total variation. Therefore, 30 repetitions of the structures shown in Fig. 1 were introduced for different wavelength width spanning from 410 nm to 450 nm to verify the robustness and universality of the proposed method.

**Fig. 5:**
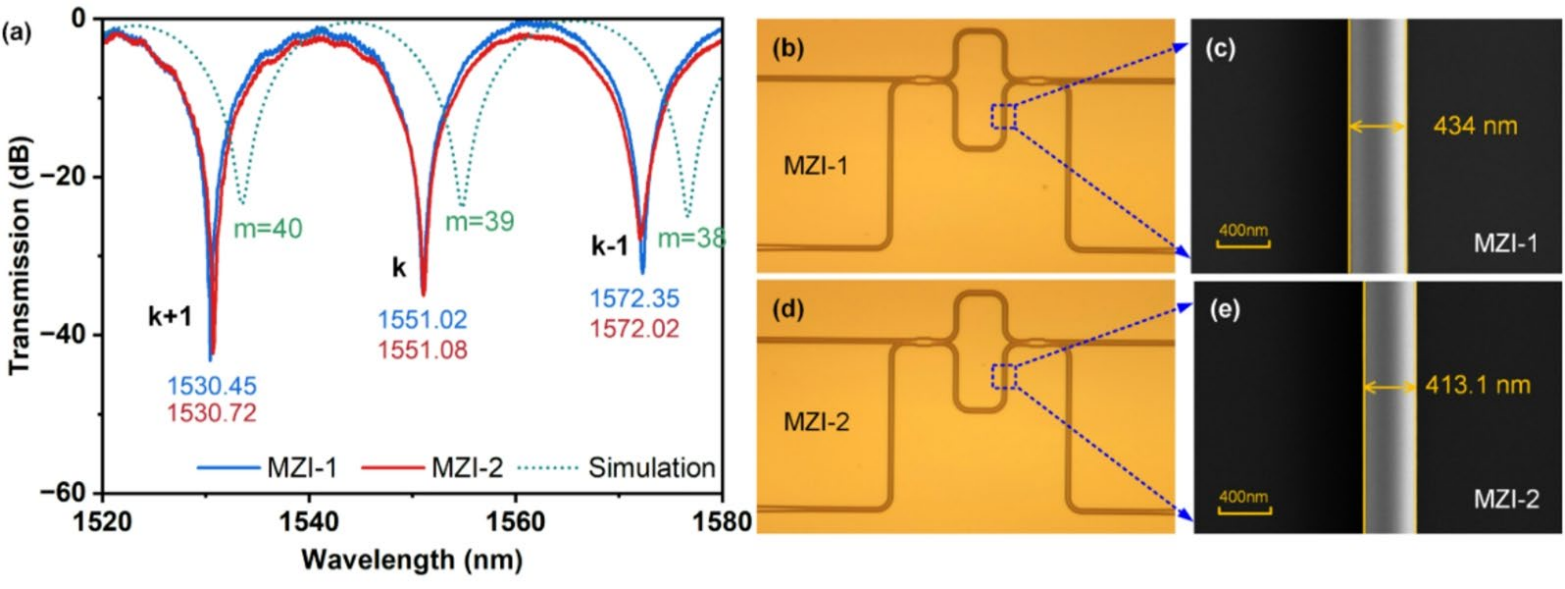
(**a**) Measured transmission patterns from MZI-1 and MZI-2 compared against simulations of the reference MZI device. Optical microscope images of the fabricated (**b**) MZI-1 and (**d**) MZI-2. SEM images of the waveguide related to (**c**) MZI-1 and (**e**) MZI-2.

**Fig. 6:**
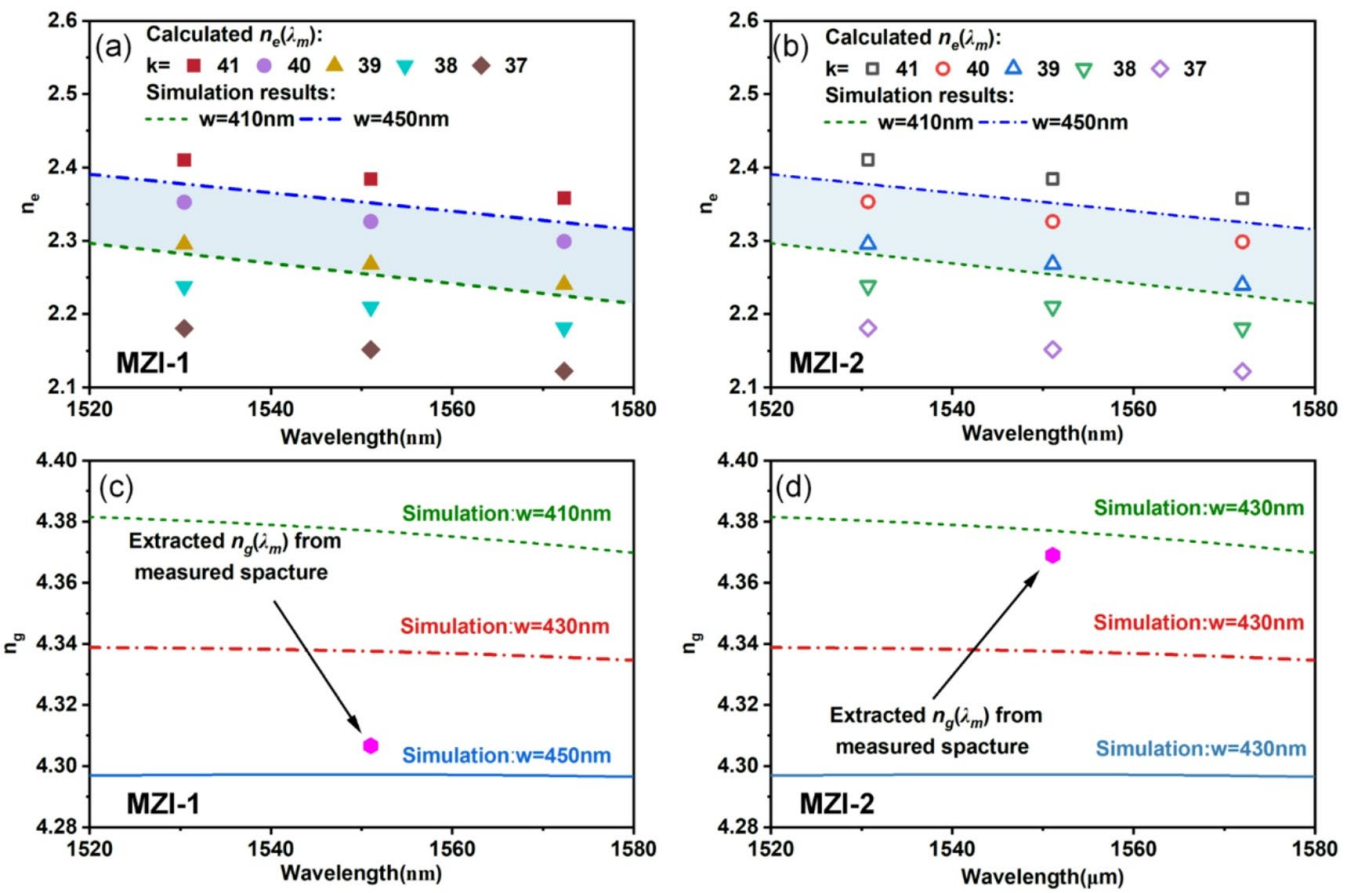
Simulated *n*_*e*_ and the calculated values of *n*_*e*_*(λ*_*des*_*)* at different values of *k* for (**a**) MZI-1 and (**b**) MZI-2. The measured values of *n*_*g*_ and the curves of simulated *n*_*g*_ for (**c**) MZI-1 and (**d**) MZI-2.

An optical spectrum analyzer that ran in tandem with a tunable laser with a central wavelength of 1550 nm was used to test the devices response. The devices transmission is normalized by that of a simple straight waveguide to eliminate the grating coupler spectrum. In order to reduce the noise in trough detection and FSR calculation, the measured transmission spectrum was preprocessed by Savitzky-Golay soomthing. Figure 5(a) compares the experimental spectra of two representative MZIs with the simulated transmission of the reference MZI (*w* = 430 nm). As demonstrated in the measured spectra, MZI-1 and MZI-2 exhibit adjacent troughs at 1551.02 nm and 1551.08 nm within the C-band. The overall morphology of the fabricated MZI-1 and MZI-2 devices is captured in the optical microscope images presented in Figs. 5(b) and (d), respectively. To validate the extracted waveguide dimensions, corresponding top-view SEM images of the waveguide sections are provided in Figs. 5(c) and (e). These close-up SEM views include direct annotations of the measured widths, offering a clear visual benchmark for comparison with the optically derived results. We define the order of the trough within the C-band as *k*. Thus, according to Eq. (2), the calculated values of *n*_*e*_*(λ*_*des*_*)* at different values of *k* for MZI-1 and MZI-2 for each troughs’ wavelength can be obtained, which can be shown in Figs. 6(a) and (b). Considering a fabrication tolerance of ± 20 nm in waveguide width, we simulated *n*_*e*_*(λ*_*des*_*)* for *w* = 410 nm and *w* = 450 nm and define the boundary limits of *n*_*e*_ region for waveguides fabricated together on the chip, as shown as the blue shaded region in the Figs. 6(a) and (b). For both MZIs, the derived *n*_*e*_*(λ*_*des*_*)* values at *k* = 39 and *k* = 40 fall within this regime.

To determine the correct and unique order of the spectrum troughs, the group refractive index of the waveguide is studied. Despite the spectral proximity, the measured spectra of the two MZIs reveal a slight disparity in their FSR. According to Eq. (3), the group index *n*_*g*_ was calculated from trough’s FSR and wavelength. As shown in Figs. 6(c)-(d), MZI-1 exhibits *n*_*g*_(1551.02 nm) = 4.307, which lies below the simulated *n*_*g*_*(λ*_*des*_*)* curve for *w* = 430 nm. This downward deviation suggests a width overshoot (*Δw* > 0), identifying its interference trough as a redshifted 40th-order interference. Conversely, MZI-2 demonstrates *n*_*g*_(1551.08 nm) = 4.369, positioned above the reference curve, confirming a width undershoot (*Δw* < 0) associated with a blueshifted 39th-order interference.

By resolving the order ambiguity, (*m* = 40 for MZI-1, *m* = 39 for MZI-2), we calculated the effective indices as *n*_*e*_(1551.02 nm) = 2.326 and *n*_*e*_(1551.08 nm) = 2.268. Feeding these wavelength dependent *n*_*e*_*(λ)* values into our pre-trained DNN, we extracted predicted waveguide widths of 438.8 nm for MZI-1 and 415.0 nm for MZI-2. Cross-validation with SEM are performed as shown in Figs. 5(c) and (e), the measurements of the actual waveguides yielded widths of 434.0 nm (MZI-1) and 413.1 nm (MZI-2), showing deviations of -4.8 nm and + 1.9 nm from the DNN predictions respectively. These errors likely originate from two primary sources: inherent process variations in maintaining width uniformity during fabrication, and unaccounted geometric parameters in our spectral analysis model, particularly sidewall angle and surface roughness.

**Fig. 7:**
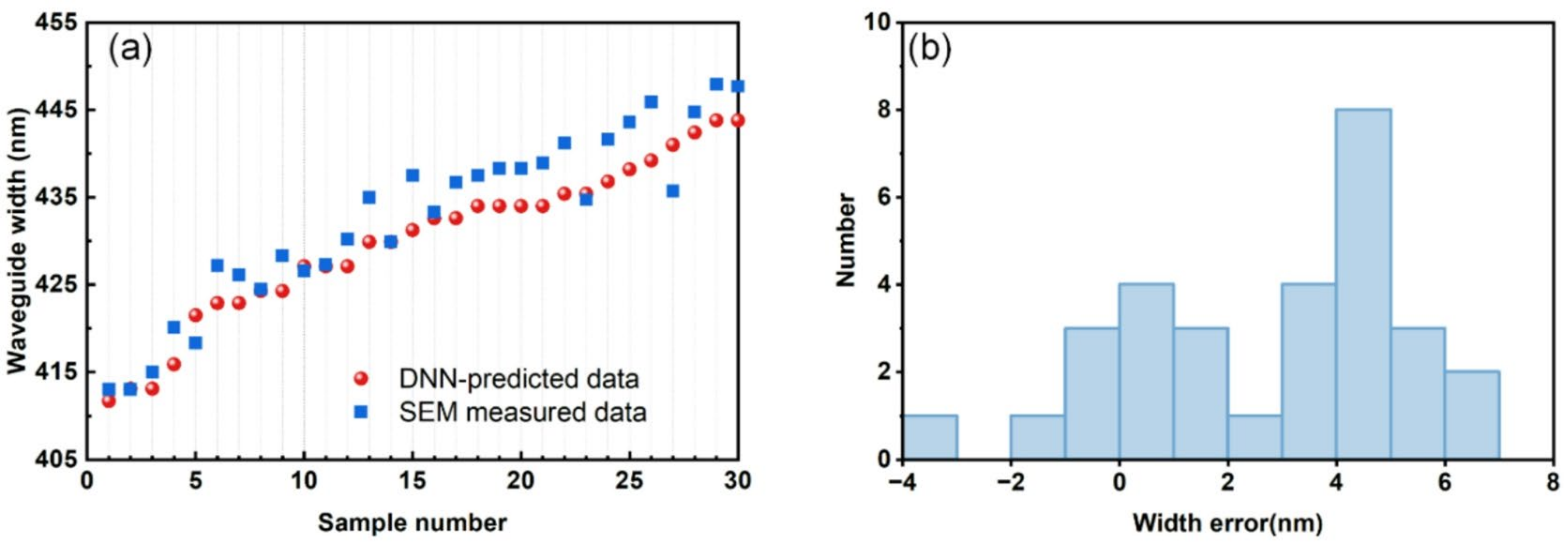
(**a**) The comparison between DNN-predicted and SEM measured waveguide width for all fabricated devices. (**b**) The distribution of their deviations.

**Table 1:** Comparative analysis with prior MZI-based waveguide characterization methods.

Ref.	Error benchmark	δw_max_ (nm)	δw_min_ (nm)	MAE (nm)		ΔL (µm)
^22^	Designed value	30	− 15	/	172	
^23^	Designed value	7.87	− 0.9	4.42	100	
This work	SEM measurement	6.7	− 3.2	3.28	26.672	

To evaluate the statistical robustness and experimental accuracy of our method, we applied the spectral analysis to all 30 fabricated devices. The overall experimental estimation error is evaluated by comparing the DNN-predicted widths against direct SEM measurements, as shown in Fig. 7(a). The deviations, plotted in Fig. 7(b), show a maximum observed discrepancy below 6.7 nm and a MAE of 3.28 nm. This overall experimental accuracy of 3.28 nm encompasses the DNN model error along with practical factors such as spectral measurement uncertainties, fabrication imperfections and the precision of the SEM reference measurement itself. The experimental data (optical transmission spectra and SEM measurements) supporting the findings of this study are available from the corresponding author upon request.

Table 1 provides an overview of the comparisons with different methods to extract the waveguide width. Unlike prior studies that measure width deviations from designed values^22,23^– a hybrid of fabrication tolerance and extraction error – our method directly compares extracted widths with SEM-measured physical dimensions. It can be seen that DNN-assisted as-fabricated waveguide width extraction achieves a discrepancy range of -3.2 nm to 6.7 nm with a MAE of 3.28 nm. Notably, while conventional techniques require MZI configurations with arm-length differences exceeding 100 μm, our method employs a single MZI device with a drastically reduced arm-length difference of 26.672 μm. This miniaturization inherently reduces device footprint, significantly enhancing photonic integration density. These advancements collectively provide a practical and compact strategy for precision metrology in nanophotonic circuit characterization.

### Error analysis

**Fig. 8:**
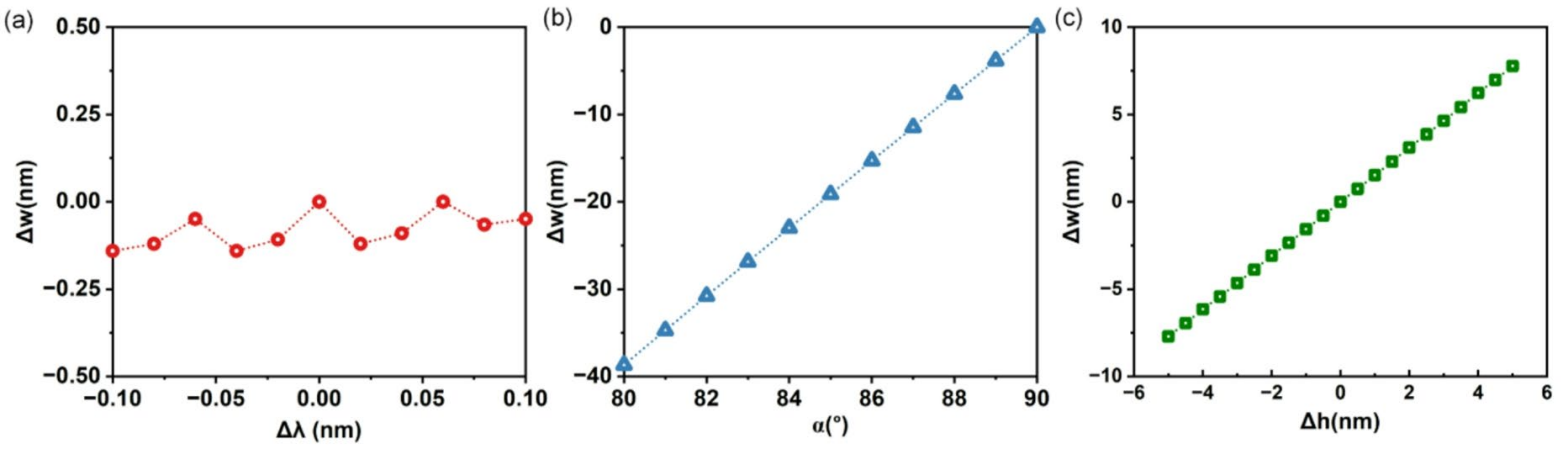
The extracted waveguide width deviation (*Δw*) in relation to (**a**) wavelength localization uncertainty (*Δλ*), (**b**) sidewall angle (*α*), and (**c**) waveguide height variation (*Δh*).

To evaluate the robustness and practical accuracy of our waveguide width extraction methodology, a comprehensive error analysis is presented, encompassing measurement uncertainties, DNN model generalization, and systematic fabrication deviations. Figure 8 illustrates the extracted waveguide width deviation (*Δw*) in relation to three key fabrication parameters: (a) the wavelength localization uncertainty (*Δλ*), (b) the sidewall angle (*α*), and (c) the variation in waveguide height (*Δh*).

1. Measurement uncertainties.

SEM metrology uncertainty: Based on repeated SEM measurements of calibration structures, we estimate the standard uncertainty in width measurement to be ± 1.5 nm (3σ confidence), arising from edge detection variability and instrument resolution.

Wavelength localization uncertainty: Within our optical measurement, the dominant uncertainty stems from the wavelength resolution of the optical spectrum analyzer (20 pm in our setup). As analyzed in Fig. 8(a), a wavelength localization error of ± 0.1 nm induces a width deviation below 0.14 nm, which is significantly smaller than the fabrication tolerance. Notably, our method exhibits strong resilience to noise because discrete trough wavelength positioning is less susceptible to random fluctuations and baseline drift compared to full-spectrum fitting techniques.

FSR uncertainty: The requirement for *n*_*g*_ is substantially relaxed, as it is used solely for resolving the interference-order ambiguity. Our analysis shows that correct order discrimination is achieved for a broad range of FSR values: for *m* = 40, FSR within [20.766, 20.961] nm, and for *m* = 39, FSR within [20.578, 20.766] nm (Fig. 6(c-d)), corresponding to an *n*_*g*_ tolerance exceeding ± 0.02.

2. DNN model generalization.

The pre-trained deep neural network achieves an MAE of 0.15 nm for width prediction on the test dataset. This error primarily originates from the model’s generalization capability and represents the fundamental precision limit of the learned *n*_*eff*_*(λ)*-to-*w* mapping under ideal conditions.

3. Model and Systematic Errors.

Discrepancies between the simulation model and actual fabricated devices introduce systematic bias. The DNN was trained using simulations that assumed vertical sidewalls (90°) and a fixed silicon layer height of 220 nm. Deviations from these ideal conditions affect the extracted width. As shown in Fig. 8(b), a decrease in the sidewall angle by 1° leads to an underestimation of the extracted width by ~ 3.8 nm, constituting a major source of systematic error. Similarly, a variation in waveguide height of ± 1 nm results in a width error of roughly ± 1.5 nm (Fig. 8(c)). Given that typical fabrication processes for SOI platforms maintain tight control over layer thickness, the resulting uncertainty in width due to height non-uniformity remains relatively small compared to the dominant width variations themselves. Therefore, while height variation is acknowledged as a source of error, its impact under standard fabrication conditions is limited and does not significantly compromise the accuracy or robustness of our proposed extraction method.

This analysis confirms that our method maintains high accuracy despite practical uncertainties, underscoring its viability for fabrication monitoring and circuit characterization.

### Outlook: extension to multi-parameter extraction

The presented DNN framework, while validated for single-parameter (waveguide width) extraction, is inherently scalable to multi-parameter estimation—such as silicon height, sidewall angle, and cladding thickness—by leveraging its capacity to model complex, high-dimensional relationships.

Extension requires: (1) designing compact test structures (e.g., MZI arrays or multi-mode devices) that yield distinct optical responses for different parameters; (2) generating simulation datasets spanning the multi-dimensional fabrication tolerance space; and (3) training a DNN with multiple optical inputs to simultaneously output several geometric parameters. The main challenge lies in designing sensitive, footprint-efficient test cells and acquiring comprehensive training data. Future work will focus on implementing such integrated multi-feature structures for holistic process monitoring.

## Conclusion

We have experimentally demonstrated a groundbreaking method combining DNNs with compact interferometers to extract waveguide widths with high accuracy. By analyzing the spectrum of C-band interference troughs and utilizing a pre-trained DNN (achieving a model mapping accuracy of 0.15 nm), our approach achieves waveguide width extraction with an experimental estimation accuracy of 3.28 nm relative to SEM standards. The system works with a single low-order MZI featuring a reduced arm-length difference of 26.672 μm, which significantly more compact than traditional designs (> 100 μm). Our method simultaneously addresses two critical challenges: accurately measuring tiny details in photonic components and making photonic circuits smaller for advanced applications. This advancement improves measurement precision and supports the development of next-generation high-density photonic chips.

## Data Availability

The datasets used for graphs and table are available from the corresponding author upon request.

## References

[1] Ahmed, A. H., Sharkia, A., Casper, B., Mirabbasi, S. & Shekhar, S. Silicon-photonics microring links for datacenters—challenges and opportunities. *IEEE J. Sel. Top. Quantum Electron.* **22**, 194–203. 10.1109/JSTQE.2016.2582345 (2016).

[2] Rizzo, A. et al. Petabit-scale silicon photonic interconnects with integrated kerr frequency combs. *IEEE J. Sel. Top. Quantum Electron.* **29**, 1–20. 10.1109/JSTQE.2022.3197375 (2023).

[3] Cheng, Q., Bahadori, M., Glick, M. & Rumley, S. Bergman. Recent advances in optical technologies for data centers: a review. *Optica* **5**, 1354–1370. 10.1364/OPTICA.5.001354 (2018).

[4] Wade, M. et al. TeraPHY: A chiplet technology for low-power, high-bandwidth in-package optical I/O. *IEEE Micro*. **40**, 63–71. 10.1109/MM.2020.2976067 (2020).

[5] Takeda, S. & Furusawa, A. Toward large-scale fault-tolerant universal photonic quantum computing. *APL Photon*. 10.1063/1.5100160 (2019).

[6] Xing, Y., Dong, J., Dwivedi, S., Khan, U. & Bogaerts, W. Accurate extraction of fabricated geometry using optical measurement. *Photon Res.* **6**, 1008–1020. 10.1364/PRJ.6.001008 (2018).

[7] Lee, J. S., Kim, Y. J., Cho, S. H., Park, B. T. & Choi, W. J. Choi. Stadium-type resonator sensor based on a multi-mode waveguide with mode discrimination phenomenon. *Opt. Express*. **31**, 19843–19852. 10.1364/OE.489554 (2023). 37381391 10.1364/OE.489554

[8] Ayotte, N., Simard, A. D. & LaRochelle, S. Long integrated bragg gratings for soi wafer metrology. *IEEE Photonics Technol. Lett.* **27**, 755–758. 10.1109/LPT.2015.2391174 (2015).

[9] Gallacher, K. et al. Characterization of integrated waveguides by atomic-force-microscopy-assisted mid-infrared imaging and spectroscopy. *Opt. Express*. **28**, 22186–22199. 10.1364/OE.393748 (2020). 32752485 10.1364/OE.393748

[10] Chen, X., Li, Z., Mohamed, M., Shang, L. & Mickelson, A. R. Parameter extraction from fabricated silicon photonic devices. *Appl. Opt.* **53**, 1396–1405. 10.1364/AO.53.001396 (2014). 24663369 10.1364/AO.53.001396

[11] Liao, J. et al. Measurement of the effective refractive index of silicon-on-insulator waveguide using Mach–Zehnder interferometers. *Sens. Actuators A: Phys.* **379**, 115906. 10.1016/j.sna.2024.115906 (2024).

[12] Zhang, E., Zhu, X. & Zhang, L. Effective and group refractive index extraction and cross-sectional dimension estimation for silicon-on-insulator rib waveguides. *Opt. Express*. **32**, 31375–31388. 10.1364/OE.534015 (2024). 39573274 10.1364/OE.534015

[13] Dwivedi, S. et al. Experimental extraction of effective refractive index and thermo-optic coefficients of silicon-on-insulator waveguides using interferometers. *J. Lightwave Technol.* **33**, 4471–4477. 10.1109/JLT.2015.2476603 (2015).

[14] Liu, D., Tan, Y., Khoram, E. & Yu, Z. Training deep neural networks for the inverse design of nanophotonic structures. *ACS Photonics*. **5**, 1365–1369. 10.1021/acsphotonics.7b01377 (2018).

[15] Ma, Z. & Li, Y. Parameter extraction and inverse design of semiconductor lasers based on the deep learning and particle swarm optimization method. *Opt. Express*. **28**, 21971–21981. 10.1364/OE.389474 (2020). 32752467 10.1364/OE.389474

[16] Wang, D. et al. System impairment compensation in coherent optical communications by using a bio-inspired detector based on artificial neural network and genetic algorithm. *Opt. Commun.* **399**, 1–12. 10.1016/j.optcom.2017.04.050 (2017).

[17] Krause, E. E. & Malka, D. Optimizations of double titanium nitride thermo-optic phase-shifter heaters using SOI technology. *Sensors* **23**, 8587. (2023). 10.3390/s23208587 37896680 10.3390/s23208587 PMC10610627

[18] Moshaev, V., Leibin, Y. & Malka, D. Optimizations of Si PIN diode phase-shifter for controlling MZM quadrature bias point using SOI rib waveguide technology. *Opt. Laser Technol.* **138**, 106844. 10.1016/j.optlastec.2020.106844 (2021).

[19] Sitbon, E., Ostrovsky, R. & Malka, D. Optimizations of thermo-optic phase shifter heaters using doped silicon heaters in Rib waveguide structure. *Photonics Nanostruct. Fundam Appl.* **51**, 101052. 10.1016/j.photonics.2022.101052 (2022).

[20] Zhang, N. M. Y. et al. Highly sensitive gas refractometers based on optical microfiber modal interferometers operating at dispersion turning point. *Opt. Express*. **26**, 29148–29158. 10.1364/OE.26.029148 (2018). 30470081 10.1364/OE.26.029148

[21] Xia, F. & Zhao, Y. RI sensing system with high sensitivity and large measurement range using a microfiber MZI and a photonic crystal fiber MZI. *Measurement* **156**, 107603. 10.1016/j.measurement.2020.107603 (2020).

[22] Oton, C. J. et al. Silicon photonic waveguide metrology using Mach-Zehnder interferometers. *Opt. Express*. **24**, 6265–6270. 10.1364/OE.24.006265 (2016). 27136819 10.1364/OE.24.006265

[23] Liu, Y., Khan, U. & Bogaerts, W. Accurately extracting silicon waveguide dimensions from a single high-order Mach-Zehnder Interferometer. *Opt. Express*. **33**, 13530–13546. 10.1364/OE.558406 (2025). 40798164 10.1364/OE.558406

